# The poor prognosis and influencing factors of high D-dimer levels for COVID-19 patients

**DOI:** 10.1038/s41598-021-81300-w

**Published:** 2021-01-19

**Authors:** Xiaokang He, Fei Yao, Jie Chen, Yan Wang, Xiangming Fang, Xuan Lin, Hui Long, Qiang Wang, Qingming Wu

**Affiliations:** 1 Tianyou Hospital, Wuhan University of Science and Technology, Wuhan, 430064 China; 2 Institute of Infection, Immunology and Tumor Microenvironment, Hubei Province Key Laboratory of Occupational Hazard Identification and Control, Medical College, Wuhan University of Science and Technology, Wuhan, 430065 China; 3 Department of Gastroenterology, Puren Hospital, Wuhan University of Science and Technology, Wuhan, 430081 China; 4 China Resources & WISCO General Hospital, Wuhan, 430081 China

**Keywords:** Biomarkers, Diseases

## Abstract

To explore the value, and influencing factors, of D-dimer on the prognosis of patients with COVID-19. A total of 1,114 patients with confirmed COVID-19 who were admitted to three designated COVID-19 hospitals in Wuhan, China from January 18, 2020, to March 24, 2020, were included in this study. We examined the relationship between peripheral blood levels of D-dimer, and clinical classification and prognosis, as well as its related influencing factors. D-dimer levels were found to be related to the clinical classification and the prognosis of clinical outcome. D-dimer levels were more likely to be abnormal in severely and critically ill patients compared with mild and ordinary cases, while D-dimer levels of patients who had died were significantly higher than those of surviving patients according to the results of the first and last lab tests. The results from ROC analyses for mortality risk showed that the AUCs of D-dimer were 0.909, YI was 0.765 at the last lab test, and a D-dimer value of 2.025 mg/L was regarded to be the optimal probability cutoff for a prognosis of death. In addition, we found that patients with advanced age, male gender, dyspnea symptoms, and some underlying diseases have a higher D-dimer value (p < 0.05). In short, D-dimer is related to the clinical classification and can be used to evaluate the prognosis of COVID-19 patients. The D-dimer value of 2.025 mg/L was the optimal probability cutoff for judging an outcome of death. Advanced age, male gender, dyspnea symptoms, and some underlying diseases are influencing factors for D-dimer levels, which impacts the prognosis of patients.

## Introduction

Corona Virus Disease 2019 (COVID-19), caused by the new coronavirus (SARS-CoV-2), is spread mainly via respiratory droplets and close contact with highly infectious people^1^. COVID-19 primarily causes lung injury, and most patients have a good prognosis, but the condition of some patients with severe infections and who are critically ill, worsens rapidly, resulting in coagulation dysfunction and even death^2,3^. Therefore, an early identification of the possible prognosis is very important for the clinical diagnosis and treatment of the COVID-19 patients. In a previous single-center, prospective study of COVID-19 patients, we observed the dynamic changes of in the peripheral blood coagulation function indices: D-dimer (DD); prothrombin time (PT); activated partial thromboplastin time (APTT); and fibrinogen (Fg). We found that D-dimer levels in particular, could be used to predict the severity and prognosis of COVID-19^4^. Based on this, we expanded the scope of our sample collection, conducted a multi-center and retrospective analysis, and further systematically studied the value and influencing factors of using D-dimer levels in the clinical classification and prognosis of COVID-19 patients.

## Materials and methods

### Source of patients and diagnosis criteria

We conducted a retrospective study focusing on the significance of D-dimer in evaluating the severity and prognosis of COVID-19. A total of 1,114 COVID-19 patients with a positive nucleic acid test for SARS-CoV-2 were collected from the COVID-19 designated hospitals in Wuhan, Hubei Province, China, including Tianyou Hospital Affiliated to Wuhan University of Science and Technology, Puren Hospital Affiliated to Wuhan University of Science and Technology, and China Resources & WISCO General Hospital between January 18, 2020, and March 24, 2020. Among them, 115 patients with COVID-19 confirmed in Tianyou Hospital have been reported in our previous study. Pregnancy patients were excluded from the study. The study was approved by the Medical Ethics Review Board of Wuhan University of Science and Technology (No. 202009), all patients involved in this study were fully informed and each provided a written informed consent. In addition, written informed consent was obtained from the legally authorized representatives or next of kin of the patients who had died. The patient data used in this study contained no personal or identifying information. All methods were performed in accordance with the relevant guidelines and regulations.

### Case classification

According to the COVID-19 diagnosis and treatment guidelines (seventh version) issued by the National Health Commission of China, COVID-19 patients are divided into four categories: mild; ordinary; severe; and critical. For our research purposes, we combined the mild and ordinary categories into a single mild or ordinary type.

### Outcome of illness

Illness outcomes were divided into: hospital discharge; improved; exacerbation; and death, according to clinical progression. We combined hospital discharge, improved and exacerbation into survival for the purpose of this study.

### Experimental data collection

A retrospective analysis of the COVID-19 patients admitted from between January 18, 2020 to and March 24, 2020 was undertaken. The D-dimer values were determined using D-dimer Assay kits (Long Island Biotech, Shanghai, China) by automatic blood coagulation analyzer mindray ExC810 (Shenzhen, China) according to the operating manual. The first D-dimer test results were obtained at admission or within 1–3 days of hospitalization, and the last D-dimer test results were from before discharge or death, or from the last test during hospitalization.

### Statistical methods

SPSS software (SPSS 24.0 for Windows, IBM, Chicago, IL, USA) was used for statistical analysis, and quantitative variables were expressed as the mean plus standard deviation (SD). Comparison between groups were performed using a t test and Mann Whitney nonparametric U test or Kruskal–Wallis H-test. The differences between groups of enumeration data were analyzed using a chi-square test. The Receiver Operating Characteristic curve (ROC) was used to calculate the area under the D-dimer curve, to evaluate the sensitivity and specificity in predicting mortality and hospital discharge. p < 0.05 was considered statistically significant.

### Ethical approval

This study was approved by the Medical Ethics Review Board of Wuhan University of Science and Technology (No. 202009).

### Consent to participate

Informed consent was obtained from all patients to be included in the study.

### Consent for publication

All authors approved the manuscript and gave their consent for publication.

## Results

A total of 1,114 cases were finally selected in this study. 885 cases and 471 cases were tested for D-dimer at the first and last lab test, respectively.

### Demographic characteristics and clinical symptoms of D-dimer patients for the first detection.

Table 1 lists the general demographic characteristics and the clinical symptoms of the 885 COVID-19 patients who received a D-dimer test as part of the initial lab test. Most patients with abnormal D-dimer values were over 60 years old and have a higher average age (p < 0.001). There was no significant difference in gender and length of hospital stay. The clinical symptoms presented by patients at admission were mainly fever (73.4%) and cough (67.0%). Patients with abnormal D-dimer values were more likely to have clinical manifestations of dyspnea, gastrointestinal and mental symptoms (p < 0.05), and to have some previous underlying condition, such as hypertension, renal insufficiency, cerebrovascular disease, or traumatic fracture (p < 0.05).

**Table 1:** General demographic characteristics and clinical manifestations of 885 COVID-19 patients with D-dimer (first lab test).

Demographic	N (%)	D-dimer < 0.5 mg/L	D-dimer ≥ 0.5 mg/L	P
Total	885 (100.0%)	556	329	
**Age (years)**				
≤ 60	427 (48.2%)	326	101	< 0.001
> 60	458 (51.8%)	230	228	
Average age (${\overline{\text{x}}} \pm {\text{s}}$)	58.83 ± 14.77	55.73 ± 14.48	64.08 ± 13.77	< 0.001
**Sex**				
Male	431 (48.7%)	272	159	0.87
Female	454 (51.3%)	284	170	
Average hospital stay (days)	16.16 ± 7.89	16.00 ± 7.71	16.47 ± 8.19	0.37
**Clinical manifestations**				
Fever	650 (73.4%)	409	241	0.92
Cough	593 (67.0%)	381	212	0.21
Chest tightness	235 (26.6%)	150	85	0.71
Difficulty breathing	233 (26.3%)	131	102	< 0.05
Gastrointestinal symptoms	64 (7.2%)	49	15	< 0.05
Spiritual consciousness	8 (0.9%)	1	7	< 0.05
**Past medical history**				
Hypertension	288 (32.5%)	160	128	< 0.05
Coronary heart disease	72 (8.1%)	39	33	0.11
Diabetes	97 (11.0%)	56	41	0.27
Renal insufficiency	20 (2.3%)	6	14	< 0.05
Cerebrovascular disease	53 (6.0%)	18	35	< 0.001
Malignant tumor	23 (2.6%)	16	7	0.50
Surgical history	86 (9.7%)	60	26	0.16
Traumatic fracture	16 (1.8%)	6	10	< 0.05

Normal range of D-dimer value: <  < 0.5 mg/L.

### The relationship between D-dimer level and clinical classification

There were significant differences in the first and last D-dimer test results for the different clinical classifications (p < 0.001). As shown in Table 2, D-dimer values were more likely to be abnormal in patients with severe and critical cases, than for those with mild or ordinary cases.

**Table 2:** Relationship between D-dimer value and clinical classification.

Clinical classification	The first test			The last test		
	N	D-dimer < 0.5 mg/L	D-dimer ≥ 0.5 mg/L	N	D-dimer < 0.5 mg/L	D-dimer ≥ 0.5 mg/L
Mild and ordinary	564	392	172	273	163	110
Severe	285	149	136	132	47	85
Critical	36	15	21	66	5	61
Total	885	556	329	471	215	256
χ^2^/p	χ^2^ = 31.24 P < 0.001			χ^2^ = 65.67 P < 0.001		

### The relationship between D-dimer level and disseminated intravascular coagulation

We explored the relationship between D-dimer levels and patients that meet ISTH criteria for disseminated intravascular coagulopathy (DIC). As shown in Table 3, the D-dimer levels of COVID-19 patients with DIC who met the ISTH diagnostic criteria were higher than for those without DIC at the first and last lab test, and the differences were significant.

**Table 3:** Relationship between D-dimer level and disseminated intravascular coagulation.

	N	D-dimer ${\overline{\text{x}}} \pm {\text{s}}\,({\text{n}})$		t/p	H(p)
		N DIC (n)	DIC (n)		
The first test	885	0.83 ± 1.90 (873)	16.14 ± 15.32 (12)	− 3.46(< 0.001)	< 0.001
The last test	471	1.31 ± 2.50 (442)	13.26 ± 13.25 (29)	− 4.85(< 0.001)	< 0.001

### The relationship between D-dimer levels and prognosis for COVID-19 patients

The final outcomes for COVID-19 patients were divided into either survival or death. Significant differences were found between D-dimer levels and outcomes at endpoints at the first and last test (p < 0.001). In addition, D-dimer levels of patients who died were significantly higher than that of surviving patients (Table 4), indicating that D-dimer may be a predictive indicator for the prognosis of COVID-19 patients.

**Table 4:** Relationship between D-dimer levels and the progression of disease.

	N	D-dimer ${\overline{\text{x}}} \pm {\text{s}}\,({\text{n}})$		t/p	H(p)
		Survival (n)	Death (n)		
The first test	885	0.80 ± 2.08(820)	3.92 ± 8.29(65)	− 3.02(< 0.05)	< 0.001
The last test	471	1.17 ± 2.67(428)	10.74 ± 11.74(43)	− 5.33(< 0.001)	< 0.001

### The value of D-dimer to evaluate prognosis

**Figure 1:**
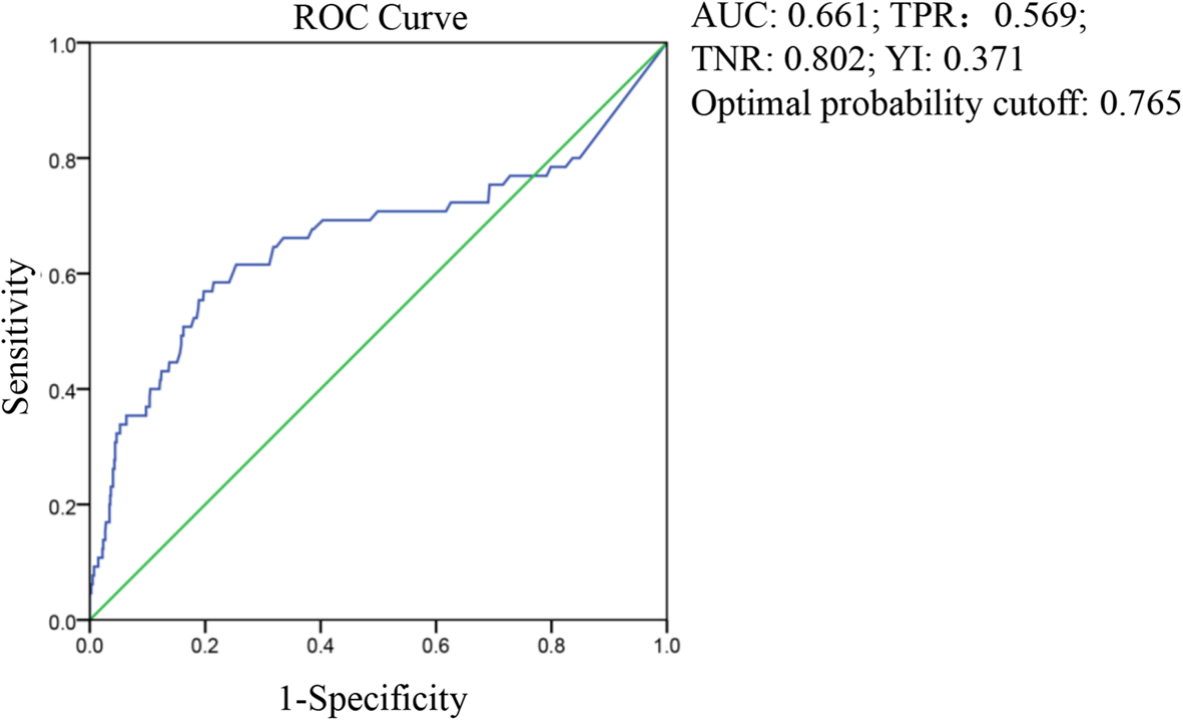
ROC curve of D-dimer from the first test, in predicting hospital discharge and mortality.

**Figure 2:**
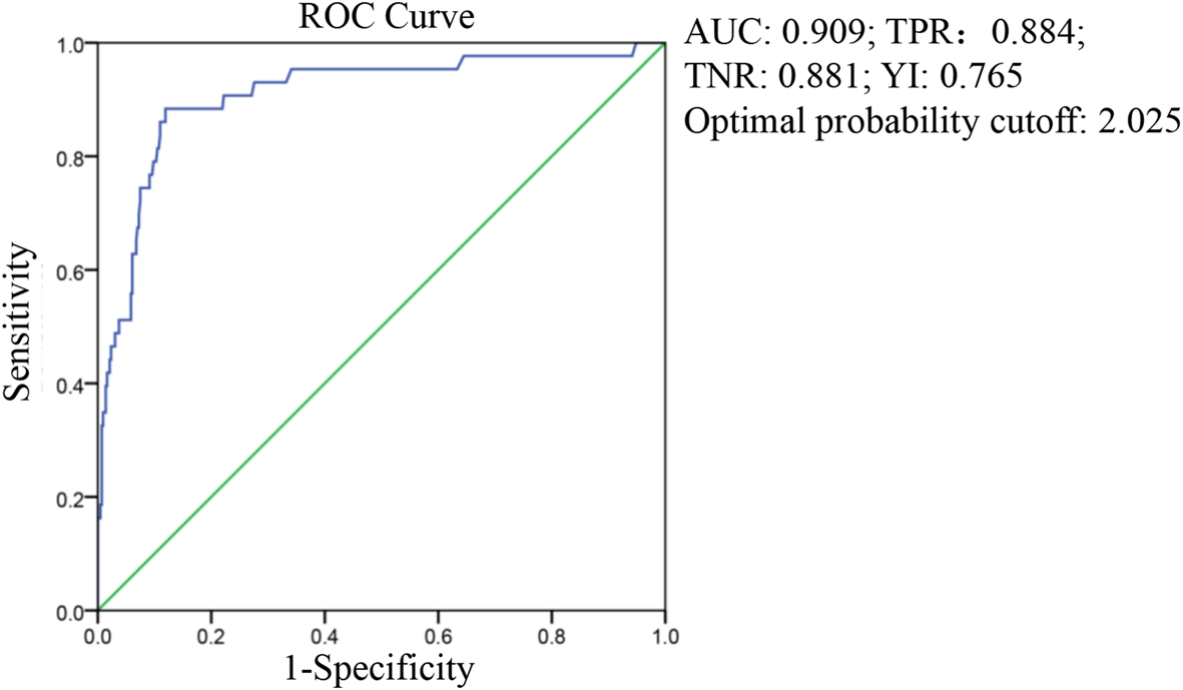
ROC curve of D-dimer from the last test, in predicting hospital discharge and mortality.

The area under the curve (AUC) of D-dimer was 0.661 at the first test, and the optimal probability cutoff was 0.765 mg/L (Fig. 1). The AUC of D-dimer was 0.909 and the optimal probability cutoff was 2.025 mg/L at the last test (Fig. 2). The last D-dimer optimal cutoff value of 2.025 mg/L was more meaningful for the prognosis of survival or death, after comparing the AUCs and Youden indices of the two curves.

### Influencing factors of D-dimer levels

The patients whose D-dimer levels were measured at the last lab test were divided into two groups with 2.025 mg/L as the critical value. The 382 patients with D-dimer levels less than the critical value were called the former group, and the 89 patients with levels greater than or equal to the critical value were called the latter group. The average age, male ratio, and proportion of patients with dyspnea or spiritual consciousness in the latter group were significantly higher. However, the number of people with spiritual consciousness in these groups was relatively small, so a larger sample is still needed to support this conclusion. Additionally, hypertension, coronary heart disease, diabetes, and cerebrovascular disease were statistically significant in both groups (Table 5). Overall, advanced age, male gender, and underlying diseases such as hypertension, coronary heart disease, diabetes, and cerebrovascular disease were influencing factors of D-dimer levels which affects the prognosis for COVID-19 patients.

**Table 5:** Influencing factors of D-dimer.

Influencing factors	N	D-dimer < 2.025 (mg/L) n = 382		D-dimer ≥ 2.025 (mg/L) n = 89		t/χ^2^ (p)	H (p)
		M ± SD	N (%)	M ± SD	N (%)		
Age (years)	471	59.53 ± 14.23	382 (100%)	68.55 ± 11.76	89 (100%)	− 6.25 (< 0.001)	< 0.001
**Sex**							
Male	242		176 (46.1%)		66 (74.2%)	22.79 (< 0.001)	
Female	229		206 (53.9%)		23 (25.8%)		
Average hospital stay (days)	471	18.13 ± 7.61	382 (100%)	18.21 ± 10.08	89 (100%)	− 0.75 (0.94)	0.65
**Clinical manifestations**							
Fever	361		290 (75.9%)		71 (79.8%)	0.60 (0.44)	
Cough	322		263 (68.8%)		59 (66.3%)	0.23 (0.64)	
Chest tightness	140		118 (30.9%)		22 (24.7%)	1.32 (0.25)	
Difficulty breathing	144		101 (26.4%)		43 (48.3%)	14.7 (< 0.001)	
Gastrointestinal symptoms	38		31 (8.1%)		7 (7.9%)	0.06 (0.94)	
Spiritual consciousness	5		2 (0.5%)		3 (3.4%)	(< 0.05)	
**Past medical history**							
Hypertension	159		114 (29.8%)		45 (50.6%)	13.86 (< 0.001)	
Coronary heart disease	46		30 (7.9%)		16 (18.0%)	8.40 (< 0.01)	
Diabetes	62		44 (11.5%)		18 (20.2%)	4.79 (< 0.05)	
Renal insufficiency	7		6 (1.6%)		1 (1.1%)	(1.00)	
Cerebrovascular disease	34		21 (5.5%)		13 (14.6%)	8.94 (< 0.01)	
Malignant tumor	13		8 (2.1%)		5 (5.6%)	3.34 (0.07)	
Surgical history	42		34 (8.9%)		8 (9.0%)	0.00 (0.98)	
Traumatic fracture	10		8 (2.1%)		2 (2.2%)	(1.00)	
Total	225		165(43.2%)		60(67.4)	16.97 (< 0.001)	

## Discussion

The clinical symptoms of COVID-19 patients were mainly fever, most being mild cases, and a few severe cases. The condition and prognosis of COVID-19 patients were complicated due to the variety of symptoms and imaging findings, and the varying degree of disease progression^4^. Notably, some severe and critical cases, as well as patients who had died, had differing degrees of coagulation dysfunction. Autopsy, and puncture histopathological observations revealed thrombus or microthrombus in the lungs, heart and liver^5–7^. In our previous study, the dynamic changes in the peripheral blood coagulation function indices (D-dimer, PT, APTT and Fg) of COVID-19 patients in a single-center and prospective study were observed, which indicated that D-dimer levels could be used for predicting the severity and prognosis of COVID-19^4^.

D-dimer is the product of fibrinolytic degradation of fibrin, and elevated levels indicate that there is a hypercoagulable state and secondary fibrinolysis in the body, which is extremely useful for the diagnosis of thrombotic diseases. Patients with COVID-19 were reported to have a hypercoagulable state^8^, with 71% of patients who died from COVID-19 were found to have met the DIC standard, this ratio among surviving patients was only 0.6%^2^. In addition, the incidence of venous thromboembolism (VTE) in patients with severe COVID-19 was 25%, and 30% of COVID-19 patients were diagnosed with pulmonary embolism^9,10^. D-dimer levels in the blood of COVID-19 patients with ischemic stroke were also increased^11^.

The results of this study showed that there were 329 (37.2%) and 256 (54.4%) patients with abnormal D-dimer values detected at the first and last test, respectively, indicating the presence of hypercoagulability in COVID-19 patients, which is consistent with the results reported above. The patients were divided into two groups based on a D-dimer value of 0.5 mg/L being the cut-off value. D-dimer values were significantly correlated with the clinical classification of the patients at the first and last test. The patients were then divided into survival and death groups according to the final outcome, and the significant differences between D-dimer and final outcomes were observed (p < 0.001). The D-dimer levels of the dead patients were significantly higher than those of surviving cases, and the D-dimer of COVID-19 patients with DIC who met the ISTH diagnostic criteria were higher than those without DIC at the first and last lab test, indicating that D-dimer levels have predictive value for the prognosis of patients. A higher D-dimer value indicated that the condition of the patient may be more serious, and these patients even combined with other serious complication. Possible reasons for increased D-dimer values for COVID-19 patients are: (1) infection can cause the release of pro-inflammatory cytokines, thus causing an inflammatory storm^12^. The levels of pro-inflammatory cytokines, such as IL-2, IL-7, G-CSF, IP-10, MCP-1, MIP-1A and TNF-α in plasma were higher especially in severe COVID-19 patients, and T cells, macrophages and natural killer cells rapidly proliferate and are highly activated, accompanied by overproduction of immune or non-immune defense cells and the release of more than 150 inflammatory cytokines and chemical mediators^13–16^. These may induce endothelial cell dysfunction, resulting in damage to the microvascular system, and abnormal activation of the coagulation system, pathological manifestations of systemic small vessel vasculitis and extensive microthrombosis^17^. (2) Some patients with COVID-19 have different degrees of hypoxia, and inflammation can lead to thrombosis or increased oxygen consumption^18^. Absolute oxygen demand increases during abnormal hemodynamics, which triggers molecular and cellular pathways and leads to thrombosis^19^. (3) Severe infection, or acute inflammation caused by sepsis, can also affect blood coagulation, such as increased levels of plasminogen activator inhibitor 1 (PAI-1), and excessive inhabited fibrinolysis^20^, which will eventually activate the coagulation cascade, and inhibit fibrinolysis as well as promoting thrombosis.

After grouping patients according to a D-dimer value of 2.025 mg/L as the critical value, we found that advanced age, male gender, dyspnea, hypertension, coronary heart disease, diabetes, and cerebrovascular disease can affect D-dimer levels and prognosis (p < 0.05). In addition, gastrointestinal and mental consciousness symptoms, renal insufficiency, and traumatic fractures were also related to D-dimer levels. Patients with mental consciousness symptoms indicated critical illness; over 50% of patients with chronic renal insufficiency already had uremia hemodialysis status. Both of these groups of patients had poor prognosis and high mortality. The COVID-19 patients with gastrointestinal symptoms and trauma fractures may have abnormal D-dimer values. D-dimer levels change with age. Male patients were more likely to have a history of smoking, which might be an influential factor. The number of patients with dyspnea was 47.2%, considering the combination of acute respiratory distress (ARDS). COVID-19 patients with hypertension and coronary heart disease might have a higher risk of death. Hypertension and COVID-19 may be related to the role of ACE2^21^. In a recent study, it was found that pericytes express high levels of ACE2 in the heart. The damage of pericytes caused by viral infections can lead to capillary vascular cell dysfunction and microvascular dysfunction^22^, which seems to explain the possible causes of acute coronary syndrome (ACS) in COVID-19 patients. Diabetes and cerebrovascular disease are risk factors affecting prognosis, COVID-19 patients with diabetes had higher D-dimer values, higher inflammatory markers, and a worse prognosis than those without diabetes^23^. In COVID-19 patients with a history of previous cerebrovascular disease (mostly cerebral infarction), there is an existing risk of atherosclerosis and emboli falling off that can lead to a hypercoagulable state. These patients also had higher levels of D-dimer. The study also showed that patients with additional underlying diseases had higher D-dimer levels, and a worse prognosis.

In summary, the results of this multi-center clinical study showed that D-dimer is related to the clinical classification of COVID-19 patients and can be used to evaluate the prognosis of patients. The D-dimer value of 2.025 mg/L is an optimal probability cutoff for judging the risk of death. After grouping according to this value, advanced age, male gender, dyspnea symptoms, and underlying diseases such as hypertension, coronary heart disease, diabetes, cerebrovascular disease became the influencing factors of D-dimer value, impacting the prognosis of patients. COVID-19 patients with the above-mentioned influencing factors have a higher risk of death. It is important to dynamically monitor D-dimer levels, to detect thrombotic complications as soon as possible, and take corresponding preventive measures to reduce thromboembolism and the risk of hemorrhage in DIC secondary fibrinolysis, thus reducing the mortality rate of COVID-19.

## Data Availability

The datasets generated and analyzed during the study are available from the corresponding author upon reasonable request.

## References

[1] Guan WJ (2019). Clinical characteristics of coronavirus disease 2019 in China. N. Engl. J. Med..

[2] Tang N, Li D, Wang X, Sun Z (2020). Abnormal coagulation parameters are associated with poor prognosis in patients with novel coronavirus pneumonia. J. Thromb. Haemost.: JTH.

[3] Zhu J (2020). Clinical characteristics of 3,062 COVID-19 patients: a meta-analysis. J. Med. Virol..

[4] Long H (2020). D-Dimer and prothrombin time are the significant indicators of severe COVID-19 and poor prognosis. Biomed. Res. Int..

[5] Fox SE (2020). Pulmonary and cardiac pathology in African American patients with COVID-19: an autopsy series from New Orleans. Lancet Respir. Med..

[6] Edler C (2020). Dying with SARS-CoV-2 infection—an autopsy study of the first consecutive 80 cases in Hamburg, Germany. Int. J. Legal Med..

[7] Lax SF (2020). Pulmonary arterial thrombosis in COVID-19 with fatal outcome: results from a prospective, single-center, clinicopathologic case series. Ann. Intern. Med..

[8] Spiezia L (2020). COVID-19-related severe hypercoagulability in patients admitted to intensive care unit for acute respiratory failure. Thromb. Haemost..

[9] Cui S, Chen S, Li X, Liu S, Wang F (2020). Prevalence of venous thromboembolism in patients with severe novel coronavirus pneumonia. J. Thromb. Haemost.: JTH.

[10] Leonard-Lorant I (2020). Acute pulmonary embolism in COVID-19 patients on CT angiography and relationship to D-Dimer levels. Radiology.

[11] Beyrouti R (2020). Characteristics of ischaemic stroke associated with COVID-19. J. Neurol. Neurosurg. Psychiatry.

[12] Chousterman BG, Swirski FK, Weber GF (2017). Cytokine storm and sepsis disease pathogenesis. Semin. Immunopathol..

[13] Chen G (2020). Clinical and immunological features of severe and moderate coronavirus disease 2019. J. Clin. Investig..

[14] Mehta P (2020). COVID-19: consider cytokine storm syndromes and immunosuppression. Lancet (London, England).

[15] Wong JP (2017). Current and future developments in the treatment of virus-induced hypercytokinemia. Future Med. Chem..

[16] Li H (2020). Serum Amyloid A is a biomarker of severe coronavirus disease and poor prognosis. J. Infect..

[17] Tian S (2020). Pulmonary pathology of early-phase 2019 novel coronavirus (COVID-19) pneumonia in two patients with lung cancer. J. Thoracic Oncol.: Off. Publ. Int. Assoc. Study Lung Cancer.

[18] Gupta N, Zhao YY, Evans CE (2019). The stimulation of thrombosis by hypoxia. Thromb. Res..

[19] Pugh CW, Ratcliffe PJ (2017). New horizons in hypoxia signaling pathways. Exp. Cell Res..

[20] Iba T (2019). Diagnosis and management of sepsis-induced coagulopathy and disseminated intravascular coagulation. J. Thromb. Haemost.: JTH.

[21] Guzik TJ (2020). COVID-19 and the cardiovascular system: implications for risk assessment, diagnosis, and treatment options. Cardiovasc. Res..

[22] Chen L, Li X, Chen M, Feng Y, Xiong C (2020). The ACE2 expression in human heart indicates new potential mechanism of heart injury among patients infected with SARS-CoV-2. Cardiovasc. Res..

[23] Guo W, Li M, Dong Y (2020). Diabetes is a risk factor for the progression and prognosis of COVID-19. Diabetes/Metab. Res. Rev..

